# Shared Gene Expression Between Multiple Sclerosis and Ischemic Stroke

**DOI:** 10.3389/fgene.2018.00598

**Published:** 2019-02-11

**Authors:** He Li, Lin Chen, Xiaofeng Ma, Pan Cui, Wenjing Lang, Junwei Hao

**Affiliations:** ^1^Department of Neurology and Tianjin Neurological Institute, General Hospital, Tianjin Medical University, Tianjin, China; ^2^Key Laboratory of Post-Neuroinjury Neuro-Repair and Regeneration in Central Nervous System, Tianjin Neurological Institute, Tianjin Medical University General Hospital, Ministry of Education and Tianjin City, Tianjin, China

**Keywords:** genome-wide association studies, multiple sclerosis, ischemic stroke, gene-based test, pathway-based analysis, histocompatibility complex variants, single nucleotide polymorphism

## Abstract

Patients with multiple sclerosis (MS) appear to have an increased risk of ischemic stroke (IS). Although MS and IS have very different phenotypes, gene-based and pathway-based analyses of large-scale genome-wide association studies (GWAS) have increasingly enhanced our understanding of these two diseases. Whether there are common molecular mechanisms connecting MS and IS is still unclear. Here, we describe the outcome of gene-based test and pathway-based analysis of GWAS datasets that explored potential gene expression links between MS and IS. After identifying significant gene sets individually of MS and IS, we performed pathway-based analysis in four biological pathway databases (KEGG, PANTHER, REACTOME, and WikiPathways) and GO categories. We discovered that there were 9 shared pathways between MS and IS in KEGG, 2 in PANTHER, 14 in REACTOME, 1 in WikiPathways, and 194 in GO annotations (*p* < 0.05). These results provide an improved understanding about possible shared mechanisms and treatments strategies for MS and IS. They also provide some basis for further studies of how these two diseases are linked at the molecular level.

## Introduction

Multiple sclerosis (MS) is a neurodegenerative, demyelinating disease of the nervous system that respects few demographic boundaries. It has an autoimmune basis, which leads to widespread nervous system tissue lesions and dysfunction, resulting in communication breakdown between neurons (Filippi and Rocca, 2005). As MS research has progressed, it has become clearer that environmental and genetic factors underlie the etiology of MS. The cooperation of these two factors in the etiology raises the question of whether one is more important than the other in posing risk, and whether co-morbidities elevate risk.

It is widely held that stroke is the second highest cause of mortality (GBD 2015 Dalys and Hale Collaborators, 2016; GBD 2015 Mortality and Causes of Death Collaborators, 2016). Stroke can result in damage to various brain areas, causing patients to suffer physically, mentally, and/or emotionally (Roger et al., 2011). There are two major categories of stroke: ischemic stroke (IS) and intracerebral hemorrhage (ICH) (Khan et al., 2013). One study found that 70–85% of all strokes are IS (Khan et al., 2013). And the studies of stroke genetics discovered several key variants like chromosome 9p21.3, *Notch3*, and *COL4A1* in early time (Cole and Meschia, 2011). This suggests that some of these unknown factors may have a genetic origin.

Recent genome-wide association studies (GWAS) of MS and IS revealed the respective genetic characteristics of these two diseases. Various major histocompatibility complex (MHC) variants (Moutsianas et al., 2015) and 110 non-MHC variants are related to MS susceptibility (International Multiple Sclerosis Genetics et al., 2013). In recent years, researchers identified the variants in *SLC9A9* and *NR1H3* had associations with the risk of MS (Liu et al., 2016; Zhang et al., 2018). Moreover, experts have focused research on network-based analyses of genome and protein pathways using GWAS datasets, especially those related to immune pathways (Baranzini et al., 2009). The International MS Genetics Consortium (IMSGC) has obtained enrichment results in gene ontology (GO) and KEGG databases with two large-scale MS-GWAS datasets two examples are apoptosis in GO and the JAK-STAT signaling pathway in KEGG (International Multiple Sclerosis Genetics, 2013). Liu et al. analyzed shared genetic pathways from different MS-GWAS datasets (Liu et al., 2017). In 1 KG dataset of IS, *ABO, HDAC9, PITX2*, and *ZFHX3* were found significant (Malik et al., 2016). The further GWAS research, 22 new significant loci were detected in the meta-analysis for stroke and its subtypes among multiple ancestries (Malik et al., 2018).

Some have noted that the risk of IS is increased for MS patients. For example, one cohort study showed that after adjusting for confounding variables, there was still an increased risk of stroke occurrence in an MS cohort compared to a control cohort (Tseng et al., 2015). In vascular diseases and autoimmune diseases, like MS, pathogenic factors such as endothelial dysfunction, atherosclerosis formation, anti-phospholipid antibody, and even smoking can contribute to decreased physical activity (Marrie et al., 2015). In MS, that decreased physical activity increases the risk for IS (Marrie et al., 2015). As our understanding of the immune-inflammatory response in stroke becomes more comprehensive, the link between IS and MS and the immune system becomes more apparent.

We hypothesize that identifying pathways shared by IS and MS will can be novel points to advance understanding of the relationship between IS and MS. Existing GWAS datasets give strong support for exploring the links between MS and IS in terms of SNP, gene and pathway analysis methods. Here, we conducted a gene-based test of IS (10,307 IS cases and 19,326 controls) and MS (9,772 MS cases and 17,376 controls) GWAS datasets following a pathway-based analysis. We found that MS and IS have in common 9 shared pathways in KEGG, 2 in PANTHER and 15 in REACTOME, 1 in Wiki pathways, and 194 in GO annotations. In short, we believe that these new results may represent significant steps toward defining the genetic mechanism underlying the association of IS with MS.

## Materials and Methods

### Samples

We used a large-scale MS-GWAS dataset from IMSGC, which was derived from the Wellcome Trust Case Control Consortium 2 (WTCCC2) project (International Multiple Sclerosis Genetics Consortium et al., 2011). This dataset comprises 9,772 MS cases and 17,376 controls of European descent, all the data of which were collected by 23 research groups working in 15 different countries. After subjecting the dataset to certain quality-control methods (such as Bayesian clustering and principal components analyses in sample QC and automated cluster and Beta-binomial model in SNP QC), 464,357 autosomal SNPs were available for genetic analysis (International Multiple Sclerosis Genetics Consortium et al., 2011).

For IS analyses, we obtained the IS dataset derived from the 1000G GWAS summary results of the METASTROKE collaboration (Malik et al., 2016). In the discovery phase, researchers gathered 12 case-control GWAS comprising 10,307 IS cases and 19,326 controls of Caucasian background. After quality-control by using logistic regression analysis (Traylor et al., 2012), meta-analysis resulted in 8.3 million SNPs. In the replication phase, the SNPs with *p* < 1.00E-05 were calculated with independent samples that included 13,435 cases and 29,269 controls of Caucasian descent and 2,385 cases and 5,193 controls of South Asian descent for replication. Finally, the results obtained from the two phases were subjected to final meta-analysis. The available data in our analysis were summarized from the discovery phase (Malik et al., 2016).

### Data Analysis

#### Gene-Based Test for MS and IS GWAS Datasets

We uploaded the SNPs data of MS and IS into VEGAS2 (Versatile Gene-based Association Study software) online. This approach is a more flexible method to assess individual SNPs and conduct gene-based testing (Mishra and Macgregor, 2015). VEGAS2 was used to analyze the hg19 annotated list derived from 1,000 genomes data from the University of California Santa Cruz (UCSC) Table Browser to simulate SNP correlations across the autosomes and chromosome X (Mishra and Macgregor, 2015). With this software, users have five ways to restrict the gene boundaries for SNP option: SNPs within 0kbloc, 10kbloc, 20kbloc, 50kbloc, and 0kbldbin (Mishra and Macgregor, 2015). First, the n SNPs' *p*-values are shifted to upper tail χ ^2^ statistics with one degree of freedom (df), and then are summarized to compute a gene-based test statistic that would have a χ ^2^ distribution with n df under the null hypothesis to define corresponding genes, if SNPs are in linkage equilibrium (Mishra and Macgregor, 2015). More detailed information about this process is found in Mishra and Macgregor (2015). Using SNPs from the 1000G European dataset, we chose a sub-population from all European. Our selections were based on “SNPs within a gene adding SNPs outside of the gene with *r*^2^ > 0.8 with SNPs within the gene.”

#### Pathway-Based Analysis for MS and IS Expression Datasets

We conducted pathway-based analysis using the WebGestalt database (Wang et al., 2017). Of the three well-established and complementary methods (ORA, NTA, and GSEA) supported by WebGestalt, we chose Over-Representation Analysis (ORA) for our enrichment analysis. ORA is a hypergeometric technique used to identify overrepresentation of genes of interest in related pathways. In addition to GO (Ashburner et al., 2000) and KEGG datasets (Kanehisa et al., 2004), we also selected the most up-to-date datasets (WebGestalt 2017) from Wiki Pathways (Pico et al., 2008); REACTOME (Fabregat et al., 2016); and PANTHER (Mi et al., 2016). Further, we used common and non-redundant GO annotations for more specific functions to be recognized. The *p*-value for observing over *J* disease-related genes in a pathway could be calculated using the following formula:

$$\text{P}=1-\sum_{\text{i}=0}^{J}\frac{\left(\begin{array}{c} \text{S}\\ \text{i} \end{array}\right)\left(\begin{array}{c} \text{N }-\text{ S}\\ \text{m }-\text{ i} \end{array}\right)}{\left(\begin{array}{c} \text{N}\\ \text{m} \end{array}\right)}$$

where m is the overall number of genes of interest associated with one given disease, N is the number of all reference genes, and S is the number of genes in the pathway. We chose pathways that contained 20–300 genes in order to exclude testing exceedingly narrow or broad pathways. The minimum number of genes per category was 5. The false discovery rate (FDR) method was used to correct for conducting multiple tests (Wang et al., 2017). Most importantly, we searched for shared biological pathways that are relevant to both MS and IS and that have an adjusted *p* < 0.05.

## Results

### Gene-Based Test of MS and IS GWAS Datasets

**Figure 1:**
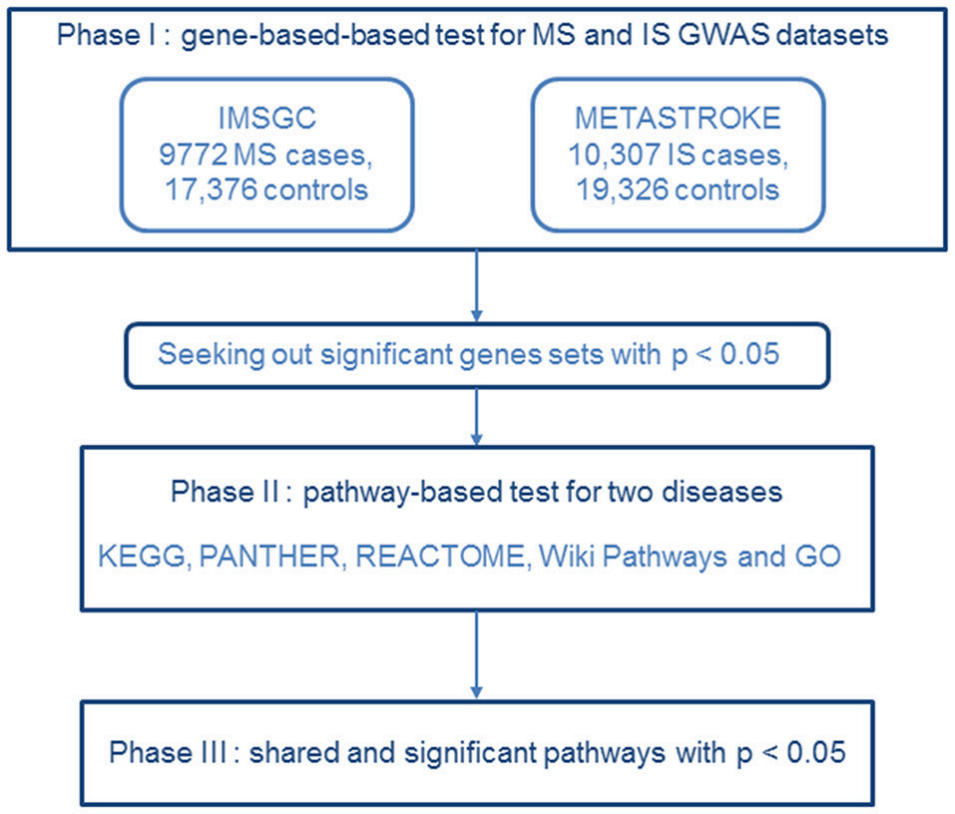
Flow diagram of the three-phase analysis design. In phase I, we performed a gene-based test using the MS dataset from IMSGC and the IS GWAS dataset from METASTROKE. Gene sets identified with *p* < 0.05 were carried forward to the next phase. In phase II, we carried out a pathway-based analysis for the two diseases. In phase III, shared and significant pathways were identified that had *p's* < 0.05.

All the 9,541,572 IS SNPs, and 464,357 MS SNPs were applied in this study. We sorted through 21,913 IS gene sets and 14,811 MS gene sets using the web-based version of VEGAS2 (Figure 1). After filtering the data for gene sets with a *p*-value of < 0.05, we obtained 1,290 IS gene sets and 1,353 MS gene sets (VEGAS2 analysis conducted 26 May 2017). The detailed results are listed in Supplementary Tables 1, 2.

### Pathway-Based Analysis of MS-GWAS Dataset

We performed pathway analysis for the 1,353 MS genes. We identified 66 significant KEGG pathways, 7 significant PANTHER pathways, 127 significant REACTOME pathways, and 58 significant Wiki Pathways (*p* < 0.05) (Supplementary Table 3). In GO, we identified 719 biological-process pathways, 170 cellular-component pathways, and 152 molecular-function pathways that had a *p*-value of < 0.05 (Supplementary Table 5).

### Pathway-Based Analysis of IS-GWAS Dataset

After uploading the 1,290 IS genes into WebGestalt database, we identified 22 significant KEGG pathways, 4 PANTHER pathways, 70 REACTOME pathways, and 13 Wiki Pathways with a *p*-value of < 0.05 (Supplementary Table 4). In Go, we identified 332 biological process pathways, 147 cellular component pathways, and 101 molecular function pathways (*p* < 0.05) (Supplementary Table 6).

### Shared Genetic Pathways Identified in MS and IS Datasets

We identified several pathways shared by MS and IS (*p* < 0.05): 9 KEGG pathways, 2 PANTHER pathways, 14 REACTOME pathways, 1 WikiPathways, and 194 GO pathways. In KEGG, the pathways fell into six categories. (1) *Immune system* (*n* = 3): natural killer cell mediated cytotoxicity (hsa04650), Toll-like receptor signaling pathway (hsa04620), and Th1 and Th2 cell differentiation (hsa04658). (2) *Environmental information processing* (*n* = 2): ErbB signaling pathway (hsa04012) and phospholipase D signaling pathway (hsa04072). (3) *Drug resistance and endocrine* (*n* = 1): EGFR tyrosine kinase inhibitor resistance (hsa01521). (4) *Nervous system* (*n* = 1): neurotrophin signaling pathway (hsa04722). (5) *Cancers* (*n* = 1): glioma (hsa05214). (6) *Infectious diseases* (*n* = 1): hepatitis B (hsa05161) (Table 1).

**Table 1:** Significant and shared KEGG pathways identified from pathway-based analysis of GWAS datasets for MS and IS.

**Classification**	**Pathway** **ID**	**Pathway name**	**IS**						**MS**					
			**C**	**O**	**E**	**R**	***p*-value**	**FDR**	**C**	**O**	**E**	**R**	***p*-value**	**FDR**
Immune system	hsa04650	Natural killer cell mediated cytotoxicity	135	16	6.87	2.33	1.30E-03	3.57E-01	135	15	8.81	1.70	2.93E-02	1.39E-01
Immune system	hsa04620	Toll-like receptor signaling pathway	106	10	5.39	1.85	4.26E-02	4.77E-01	106	14	6.91	2.02	8.62E-03	6.58E-02
Immune system	hsa04658	T1 and Th2 cell differentiation	92	9	4.68	1.92	4.36E-02	4.77E-01	92	20	6.00	3.33	1.33E-06	3.65E-04
Drug resistance	hsa01521	EGFR tyrosine kinase inhibitor resistance	81	10	4.12	2.43	7.65E-03	4.29E-01	81	13	5.28	2.46	2.06E-03	2.50E-02
Nervous system	hsa04722	Neurotrophin signaling pathway	121	13	6.16	2.11	8.27E-03	4.29E-01	121	16	7.89	2.03	5.11E-03	4.53E-02
Environmental information processing	hsa04012	ErbB signaling pathway	88	10	4.48	2.23	1.34E-02	4.29E-01	88	12	5.74	2.09	1.13E-02	7.80E-02
Cancers	hsa05214	Glioma	66	8	3.36	2.38	1.81E-02	4.29E-01	66	9	4.30	2.09	2.65E-02	1.30E-01
Environmental Information Processing	hsa04072	Phospholipase D signaling pathway	144	13	7.33	1.77	3.12E-02	4.77E-01	144	17	9.39	1.81	1.22E-02	8.10E-02
Infectious diseases	hsa05161	Hepatitis B	146	13	7.43	1.75	3.44E-02	4.77E-01	146	19	9.52	1.99	2.87E-03	3.03E-02

*GWAS, large-scale genome-wide association studies; C, the number of reference genes in the category; O, the number of genes in the gene set and also in the category; E, expected number in the category; R, the ratio of enrichment; FDR, false discovery rate; KEGG, Kyoto Encyclopedia of Genes and Genomes*.

In PANTHER, two pathways were shared: Cadherin signaling pathway (P00012) and Wnt signaling pathway (P00057). In REACTOME, 14 significant pathways were shared, for example, cell-cell communication (R-HSA-1500931) and interferon gamma signaling (R-HSA-877300) (Table 2). In WikiPathways, only one pathway was shared: thymic stromal lymphopoietin (TSLP) signaling pathway (WP2203).

**Table 2:** Results of pathway-based analysis in IS and MS GWAS datasets showing significant and shared IS-MS pathways in VEGAS in PANTHER, REACTOME and WikiPathways.

**Pathway ID**	**Description**	**IS**						**MS**					
		**C**	**O**	**E**	**R**	***p*-value**	**FDR**	**C**	**O**	**E**	**R**	***p*-value**	**FDR**
**PANTHER**													
P00012	Cadherin signaling pathway	153	22	8.80	2.50	3.34E-05	2.51E-03	153	27	12.1	2.23	3.32E-05	2.49E-03
P00057	Wnt signaling pathway	294	31	16.9	1.83	3.63E-04	1.36E-02	294	38	23.2	1.64	9.17E-04	2.29E-02
**REACTOME**													
R-HSA-8876198	RAB GEFs exchange GTP for GDP on RABs	90	12	4.51	2.66	1.72E-03	2.45E-01	90	10	5.50	1.82	4.75E-02	3.32E-01
R-HSA-1839124	FGFR1 mutant receptor activation	31	6	1.56	3.86	3.90E-03	2.45E-01	31	5	1.90	2.64	3.82E-02	2.87E-01
R-HSA-111885	Opioid signaling	85	10	4.26	2.34	9.84E-03	3.71E-01	85	10	5.20	1.92	3.40E-02	2.70E-01
R-HSA-2219530	Constitutive signaling by aberrant PI3K in cancer	65	8	3.26	2.45	1.55E-02	4.02E-01	65	10	3.97	2.52	5.76E-03	9.38E-02
R-HSA-445144	Signal transduction by L1	21	4	1.05	3.79	1.90E-02	4.20E-01	21	4	1.28	3.11	3.61E-02	2.80E-01
R-HSA-1500931	Cell-cell communication	141	13	7.07	1.84	2.47E-02	4.20E-01	141	14	8.62	1.62	4.95E-02	3.43E-01
R-HSA-6811434	COPI-dependent golgi-to-ER retrograde traffic	98	10	4.92	2.03	2.48E-02	4.20E-01	98	12	5.99	2.00	1.61E-02	1.79E-01
R-HSA-373753	Nephrin interactions	23	4	1.15	3.47	2.59E-02	4.23E-01	23	5	1.41	3.55	1.13E-02	1.48E-01
R-HSA-6811558	PI5P, PP2A and IER3 Regulate PI3K/AKT signaling	87	9	4.36	2.06	2.99E-02	4.70E-01	87	11	5.32	2.07	1.66E-02	1.79E-01
R-HSA-2219528	PI3K/AKT signaling in cancer	90	9	4.51	1.99	3.61E-02	5.28E-01	90	13	5.50	2.36	3.11E-03	5.59E-02
R-HSA-913531	Interferon signaling	199	16	9.98	1.60	4.22E-02	5.61E-01	199	21	12.17	1.73	1.01E-02	1.41E-01
R-HSA-877300	Interferon gamma signaling	93	9	4.67	1.93	4.32E-02	5.61E-01	93	15	5.69	2.64	4.80E-04	1.21E-02
R-HSA-6783589	Interleukin-6 family signaling	27	4	1.35	2.95	4.40E-02	5.61E-01	27	8	1.65	4.85	1.47E-04	5.63E-03
R-HSA-199418	Negative regulation of the PI3K/AKT network	94	9	4.72	1.91	4.58E-02	5.75E-01	94	11	5.75	1.91	2.79E-02	2.39E-01
**WIKI PATHWAYS**													
WP2203	Thymic stromal Lymphopoietin (TSLP) signaling pathway	47	7	2.60	2.69	1.39E-02	6.89E-01	47	14	3.21	4.36	1.62E-06	1.19E-04

*Same conventions as in Table 1*.

**Figure 2:**
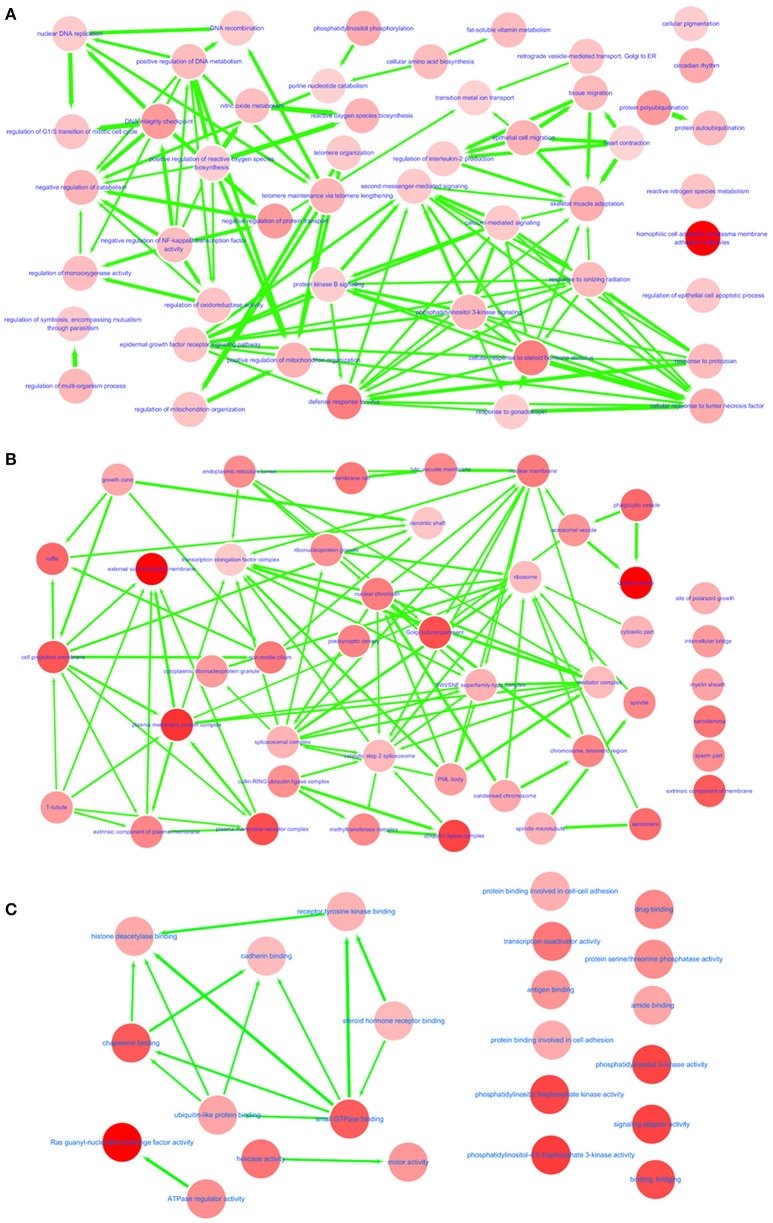
Interactive networks between MS and IS. Shared and significant biological process **(A)**, cellular component **(B)**, and molecular function **(C)** from GO function annotation. Bubble color indicates the *p*-value of each annotation; line width indicates the degree of similarity; arrows represent common affiliations between them.

**Figure 3:**
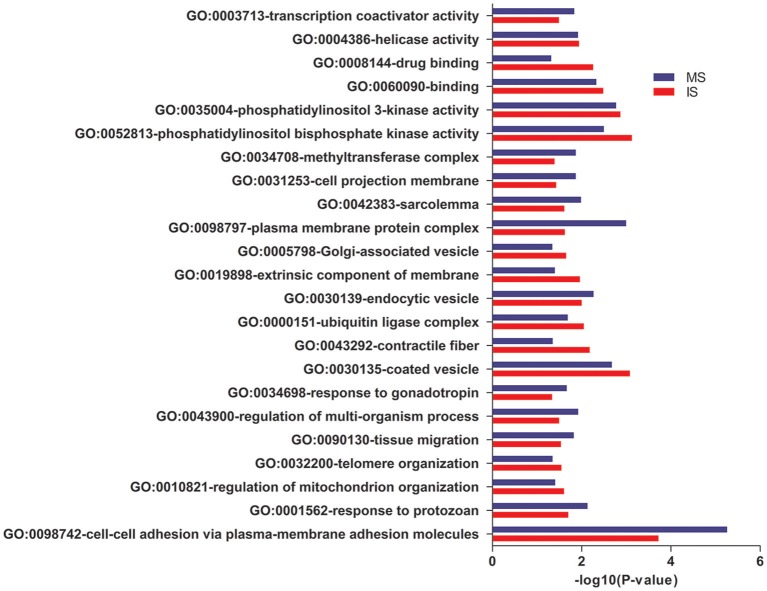
Pathway analysis. Shared and significant, non-redundant GO annotations. Enriched clusters between MS and IS, including the –log10 (*P*-value), are shown.

In GO annotations, of the 194 shared pathways identified, 85 were biological-process pathways, 78 were cellular component pathways, and 31 were molecular-function pathways. After analyzing these data using Cytoscape software (Shannon et al., 2003), we found that interactions exist between MS and IS in these three categories of pathways (Figure 2). For our comparisons to be concise, however, we needed non-redundant GO annotations. In non-redundant GO annotations, we obtained 7 biological-process pathways (GO: 0098742, 0001562, 0010821, 0032200, 0090130, 0043900, and 0034698); 10 cellular-component pathways (GO: 0030135, 0043292, 0000151, 0030139, 0019898, 0005798, 0098797, 0042383, 0031253, and 0034708); and 6 molecular-function pathways (GO: 0052813, 0035004, 0060090, 0008144, 0004386, and 0003713) (Table 3). Enriched clusters are illustrated in Figure 3.

**Table 3:** Results of from pathway-based analysis in IS and MS GWAS datasets showing significant and shared IS-MS pathways in VEGAS in non- redundant GO.

**Gene set**	**Description**	**IS**						**MS**					
		**C**	**O**	**E**	**R**	***p*-value**	**FDR**	**C**	**O**	**E**	**R**	***p*-value**	**FDR**
**BIOLOGICAL PROCESS**													
GO:0098742	Cell-cell adhesion via plasma-membrane adhesion molecules	224	25	11.40	2.19	1.89E-04	5.66E-02	224	32	13.62	2.35	5.53E-06	1.66E-03
GO:0001562	Response to protozoan	21	4	1.07	3.74	1.99E-02	4.47E-01	21	5	1.28	3.92	7.39E-03	1.20E-01
GO:0010821	Regulation of mitochondrion organization	229	19	11.65	1.63	2.48E-02	4.47E-01	229	21	13.93	1.51	3.94E-02	2.59E-01
GO:0032200	Telomere organization	127	12	6.46	1.86	2.83E-02	4.47E-01	127	13	7.72	1.68	4.51E-02	2.80E-01
GO:0090130	Tissue migration	233	19	11.86	1.60	2.90E-02	4.47E-01	233	23	14.17	1.62	1.50E-02	1.66E-01
GO:0043900	Regulation of multi-organism process	267	21	13.59	1.55	3.19E-02	4.66E-01	267	26	16.24	1.60	1.20E-02	1.49E-01
GO:0034698	Response to gonadotropin	27	4	1.37	2.91	4.60E-02	5.31E-01	27	5	1.64	3.05	2.17E-02	1.90E-01
**CELLULAR COMPONENT**													
GO:0030135	Coated vesicle	235	21	9.82	2.14	8.26E-04	4.79E-02	235	22	11.31	1.95	2.11E-03	8.14E-02
GO:0043292	Contractile fiber	209	17	8.73	1.95	6.56E-03	1.15E-01	209	16	10.06	1.59	4.45E-02	2.13E-01
GO:0000151	Ubiquitin ligase complex	287	21	12.00	1.75	8.92E-03	1.15E-01	287	22	13.81	1.59	2.06E-02	1.83E-01
GO:0030139	Endocytic vesicle	254	19	10.62	1.79	1.00E-02	1.15E-01	254	22	12.22	1.80	5.42E-03	1.05E-01
GO:0019898	Extrinsic component of membrane	256	19	10.70	1.78	1.09E-02	1.15E-01	256	19	12.32	1.54	3.99E-02	2.13E-01
GO:0005798	Golgi-associated vesicle	83	8	3.47	2.31	2.22E-02	1.65E-01	83	8	3.99	2.00	4.56E-02	2.13E-01
GO:0098797	Plasma membrane protein complex	222	16	9.28	1.72	2.37E-02	1.65E-01	222	22	10.68	2.06	1.01E-03	7.84E-02
GO:0042383	Sarcolemma	117	10	4.89	2.05	2.44E-02	1.65E-01	117	12	5.63	2.13	1.04E-02	1.58E-01
GO:0031253	Cell projection membrane	292	19	12.20	1.56	3.70E-02	1.95E-01	292	23	14.05	1.64	1.36E-02	1.58E-01
GO:0034708	Methyltransferase complex	93	8	3.89	2.06	4.03E-02	2.03E-01	93	10	4.47	2.23	1.36E-02	1.58E-01
**MOLECULAR FUNCTION**													
GO:0052813	Phosphatidylinositol bisphosphate kinase activity	65	10	3.01	3.32	7.47E-04	9.12E-02	65	10	3.65	2.74	3.18E-03	9.69E-02
GO:0035004	Phosphatidylinositol 3-kinase activity	70	10	3.24	3.09	1.35E-03	1.10E-01	70	11	3.93	2.80	1.69E-03	7.49E-02
GO:0060090	Binding, bridging	176	17	8.15	2.09	3.28E-03	2.00E-01	176	19	9.89	1.92	4.71E-03	1.05E-01
GO:0008144	Drug binding	98	11	4.54	2.43	5.51E-03	2.69E-01	98	10	5.51	1.82	4.82E-02	2.87E-01
GO:0004386	Helicase activity	153	14	7.08	1.98	1.14E-02	3.99E-01	153	16	8.60	1.86	1.21E-02	1.38E-01
GO:0003713	Transcription coactivator activity	294	21	13.61	1.54	3.22E-02	5.83E-01	294	26	16.52	1.57	1.47E-02	1.43E-01

*Same conventions as in Table 1*.

## Discussion

What accounts for the increased risk of IS in patients with MS (Tseng et al., 2015)? Here, we described, through a pathway-based analysis of IS and MS GWAS datasets, gene expression links between IS and MS. We discovered 9 shared pathways between MS and IS in KEGG, 2 in PANTHER, 15 in REACTOME, 1 in WikiPathways, and 194 in GO annotations. These results provide an improved understanding of possible shared mechanisms and treatment strategies for IS and MS.

To date, MS and IS GWAS research have identified some vital SNPs, gene sets, and pathways for the respective diseases. The most well-known associated gene is *CD40*, a member of the TNF-receptor superfamily. Several studies have reported that *CD40* and related SNPs are closely associated with MS susceptibility. Using multiple statistical analysis methods, Sokolova et al. confirmed that SNP rs6074022 located in *CD40* was related to a higher risk of MS development (Sokolova et al., 2013). Other SNPS of *CD40* (rs1883832 C/T, rs13040307 C/T, rs752118 C/T, and rs3765459 G/A; and rs1883832 C/T polymorphism and its TCCA haplotype) were suggested to be associated with IS susceptibility at a significant test threshold (Chen et al., 2016). After adjusting for relevant covariates, one clinical study reported a higher risk of IS in the MS cohort compared to the control cohort (Tseng et al., 2015). This was corroborated by findings that both young and older MS patients are at an increased risk for IS (Jadidi et al., 2013).

To convince ourselves that there is indeed a clear but subtle link between MS and IS, we focused our analysis on genes that these two diseases might share in common. In phase I, we confirmed 1,290 IS and 1,353 MS significant genes with IS-GWAS datasets and MS-GWAS. In phase II, we conducted enrichment analysis via GO, KEGG, PANTHER, Wiki Pathways, and REACTOME. In phase III, we selected shared significant pathways between the two diseases using the datasets of each disease. As we determined associations between MS and IS at the gene expression level, which was the most important goal of our research, we detected more risk pathways, like natural killer cell-mediated cytotoxicity pathways in the immune system. So far, the common treatments of MS and IS are mainly associated with the neuroinflammation for some similar pathomechanisms (Paterno and Chillon, 2018). Natalizumab (Elkins et al., 2017), minocycline (Schabitz et al., 2008), and fingolimod (Fu et al., 2014), three approved agents for MS, have been applied in clinical trials already. And in recent years more immunomodulatory drugs approved for MS became a magnet for the therapies of IS, for instance, glatiramer acetate (Poittevin et al., 2013), and DMF (Lin et al., 2016). Our findings offer innovative insights into pathway analysis, which will be crucial for deciphering the pathogenesis underlying the MS-IS relationship and for instituting appropriate therapeutic regimes.

### Shared KEGG Pathways

The association between the pathophysiology of IS and MS and how it is related to the immune system is becoming more and more clear. In the context of pathways shared by IS and MS, this association motivated us to focus our attention on key pathways in the immune system and the nervous system.

One significant shared pathway identified from our analyses is the natural killer (NK) cell-mediated cytotoxicity pathway (hsa04650). NK cell-mediated cytotoxicity is one of the main characteristics of NK cells. NK cells release cytotoxic granules onto the target cell's surface, causing effector proteins to penetrate the cell membrane and induce programmed cell death.

Over the past decades, both in clinical trials and animal experiments, NK cell dysfunction has been shown to be strongly associated with the immunopathogenesis of MS and certain patients' responses to certain treatments (Morandi et al., 2008). There are two major functional subtypes of NK cells, CD56^dim^, CD16^hi^, and CD56^bright^, the latter of which may play a key role in MS and IS immunopathology. CD56^bright^ NK cells, which are normally weakly cytotoxic, can acquire cytotoxic properties and produce cytokines if they are stimulated (Melsen et al., 2016), as, for example, with certain therapeutic agents. This increased NK activity correlates with responses to immunotherapies. For example, patients treated with daclizumab or IFNβ produce more CD56^bright^ NK cells (Bielekova et al., 2006; Saraste et al., 2007). In addition, NK cells are more cytotoxic toward autologous activated T cells in samples from patients treated with daclizumab than those from untreated patients (Jiang et al., 2011).

The role of NK cells in the pathophysiology of MS and IS was further elucidated in another study. Jiang and colleagues demonstrated that the acetylcholine-producing NK cells reduce CNS damage in an animal model of MS (Jiang et al., 2017). Natural killer cell-mediated cytotoxicity (hsa04650) also has been demonstrated the significance by pathway analysis in GWAS datasets (Giacalone et al., 2015). After acute stroke, ischemic neurons release fractalkine to recruit lymphocytes, including NK cells, to gather in the injured areas (Gan et al., 2014). There, NK cells can induce neuronal death by secreting cytokines and glutamate; this is one inflammatory mechanism that can lead to tissue damage (Gan et al., 2014). A meta-analysis of all types of stroke (IS and its subtypes) of 12 different GWAS identified nearly 100 different pathways associated with each type of stroke (Bonferroni corrected *p* < 0.05); however, only the NK cell signaling pathway was unique in that it was significantly shared by all stroke and IS subtypes (Malik et al., 2016).

Another pathway shared by MS and IS is the Toll-like receptor signaling pathway (hsa04620). The Toll-like receptor (TLR) family is a well-known class of proteins that are prototype pattern-recognition receptors (PRRs) capable of recognizing pathogen-associated molecular patterns (PAMPs), which are signature motifs possessed by certain pathogenic microorganisms; and danger-associated molecular patterns (DAMPs), which are host molecules that initiate an inflammatory response to damaged tissues or lesions (Kawasaki and Kawai, 2014). The Toll-like receptor signaling pathway (hsa04620) is complicated, as it can activate many important signaling molecules such as nuclear factor-κB (NF-κB) transcription factors, mitogen-activated protein kinases (MAPKs), and p3 (Kawasaki and Kawai, 2014). TLR signaling pathways extensively influence the immune system. Thus, it is not surprising that many diseases have multiple links with these pathways. For example, TLR4 aggravates inflammation in experimental autoimmune encephalomyelitis (EAE) model (Reynolds et al., 2012). TLR2 expression on oligodendrocytes is enhanced in MS lesions but not on oligodendrocytes in normal areas (Hanafy and Sloane, 2011).

TLR2 can also promote immune responses through Th17 cells (Reynolds et al., 2010). In the CNS, a given TLR can stimulate diverse signaling pathways in different neural cells. After a stroke, DAMPs activate TLR2 and TLR4 in microglia to increase the production of pro-inflammatory cytokines (Caso et al., 2007; Lehnardt et al., 2007). In ischemic neurons, TLR2 and TLR4 activate downstream elements, JNK and AP-1, to initiate proapoptotic activity (Tang et al., 2007). In one clinical trial, researchers discovered that TLR7 and TLR8 are related to poor outcome in IS (Brea et al., 2011). Similarly, in a mouse stroke model, TLR3 and TLR9 did not confer protection to neural cells during middle cerebral artery occlusion (Hyakkoku et al., 2010).

Yet another pathway significantly shared by MS and IS is the Th1 and Th2 cell differentiation pathway (hsa04658). Through this pathway, naïve CD4^+^ T cells respond to cues from antigen presenting cells (APC), causing them to differentiate into Th1 and Th2 cells, which are two major subtypes of effector CD4^+^ T cells. The differentiation of Th1 and Th2 cells depends on the signals they receive. Th1 cells are triggered by IL-12 and secrete IFN-γ and IL-2, whereas Th2 cells are triggered by IL-4 and IL-2, and secrete IL-4, IL-5, IL-9, IL-10, IL-13, and IL-25 (Zhu and Paul, 2008). In clinical trials, IL-17 and IFN-γ were shown to exacerbate the symptoms of MS patients; this provided evidence that Th1 and Th17 cells can have an impact on diseases through cytokines (Panitch et al., 1987; Havrdova et al., 2016). Th1 and Th2 cells exert opposite effects on infarct lesions in mice undergoing middle cerebral artery occlusion. In these mice, Th2 deficiency increased infarct size by enhancing recruitment of macrophages and neutrophils to the infarct, whereas Th1 deficiency decreased infarct size by retarding recruitment of macrophages and neutrophils to the infarct (Gu et al., 2012). Moreover, stroke initially causes a decrease in immune cells in the penumbra, leading to a shift from Th1 to Th2 cytokines which is associated with stroke-induced immunosuppression (Prass et al., 2003).

Neurotrophin signaling pathway (hsa04722) is another pathway shared by MS and IS. It influences the differentiation and survival of neural cells. Nerve growth factor (NGF), brain derived neurotrophic factor (BDNF), neurotrophin 3 (NT-3), and neurotrophin 4 (NT-4) are major members of the neurotrophin family. The Trk family of tyrosine kinase receptors and p75 neurotrophin receptors (p75NTRs) are two major neurotrophin receptors (Reichardt, 2006). BDNF/TrkB signaling and TrkB-FL/TrkB-T1 balance are two targets for stroke therapies (Vidaurre et al., 2012). Ciliary neurotrophic factor (CNTF) has been reported to have a neuroprotective effect in the cortex of MS patients (Dutta et al., 2007). Glial p75NTRs are increased during plaque formation in MS (Dowling et al., 1999).

MS and IS are diseases that involve both the immune system and CNS. Future studies should direct more attention to analyzing shared and significant pathways of the immune system and nervous system. Doing so will advance understanding of the shared mechanisms underlying the pathogenesis of these two diseases. Moreover, new information gleaned from important pathways identified in MS will suggest meaningful targets in IS to focus on and vice versa.

### Shared Gene Ontology Enrichment Analysis

We used annotations from the GO project to identify any significant relationships in biological functions encoded by shared genes at the molecular, cellular, and tissue levels. We identified 194 annotations total, spread among three functional categories: biological process, cellular component, and molecular function. It was convenient for us to study the function of the genome on a more advanced level, for example, to estimate which part of the genome is shared between diseases with regard to signal transduction, metabolism synthesis or copy number. We further narrowed down the annotations of shared genes and identified 7 in biological process, 10 in cellular component, and 6 in molecular function. Enrichment analysis identified cell-cell adhesion via plasma-membrane adhesion molecules (GO: 0098742), as one of the most significant GO functional category shared by IS and MS. Adhesion molecules of lymphocytes are significantly elevated in IS patients (Tsai et al., 2009). In MS, adhesion molecules on immune cells play a role in disease progression (Dhib-Jalbut, 2002). Targeting these adhesion molecules with glatiramer acetate has been shown to reduce the pro-migratory components in MS (Sellner et al., 2013).

#### Limitations

Despite of the novel findings, there are some limitations to this research. The GWAS datasets we chose were representative but the replications of multiple datasets should be conducted to improve the validity of the results. Next, due to the absence of IS subtypes' data, we did not analyze the associations between MS and the etiological subtypes of IS. Nevertheless, risk variants of IS are gradually identified to be related to its subtypes. At last, we have used reliable statistical methods to identify significant shared pathways after filtering out significant genes, respectively, but there is a chance that the commonalities might be found out between two diseases that are unlikely to share mechanisms. In further studies, our results need more experiments to explore and validate.

## Conclusions

We report on the significant pathways and GO annotations shared by MS and IS, with the goal of understanding more about their biological functions and relationships. By analyzing the pathways of the immune system and nervous system, we can verify that links between MS and IS exist and infer gene expression level correlations. Leveraging information about where biological functions overlap, we believe that a multidisciplinary approach will advance studies on the pathophysiological mechanisms in and treatments for both MS and IS.

## Author Contributions

HL and JH conceived and designed the study for MS and IS. HL administered the analyses and wrote the manuscript. LC and XM was responsible for manuscript revision. PC and WL provided analyses support. All authors gave approval for the final version for submission.

### Conflict of Interest Statement

The authors declare that the research was conducted in the absence of any commercial or financial relationships that could be construed as a potential conflict of interest.

## Abbreviations

CNS: central nervous system
DAMPs: danger-associated molecular patterns
EAE: experimental autoimmune encephalomyelitis
FDR: false discovery rate
GO: gene ontology
GSEA: Gene Set Enrichment Analysis
GWAS: genome-wide association study
IMSGC: International MS Genetics Consortium
IS: ischemic stroke
KEGG: Kyoto Encyclopedia
MS: multiple sclerosis
NTA: Network Topology-based Analysis
ORA: Over-Representation Analysis
PAMPs: pathogen-associated molecular patterns
PRRSs: pattern-recognition receptors
QC: quality control
SNP: single nucleotide polymorphism
TLR: Toll-like receptor (TLR)
TSLP: Thymic stromal lymphopoietin
VEGAS2: Versatile Gene-based Association Study-2 version 2
WTCCC2: Wellcome Trust Case Control Consortium 2.
